# The value of a specialized second-opinion pathological diagnosis for oral and maxillofacial lesions

**DOI:** 10.1186/s12903-023-03085-w

**Published:** 2023-06-09

**Authors:** Nada O. Binmadi, Areej A. Alhindi, Maha T. Alsharif, Basem T. Jamal, Yasmin H. Mair

**Affiliations:** 1 Department of Oral Diagnostic Sciences, King Abdulaziz University Faculty of Dentistry, P.O. Box 80209, Jeddah, 21589 Saudi Arabia; 2 Department of Oral and Maxillofacial Surgery, King Abdulaziz University Faculty of Dentistry, Jeddah, Saudi Arabia

**Keywords:** Second opinion, Consultation, Referral, Oral pathology, Diagnostic errors, Squamous cell carcinoma

## Abstract

**Objectives:**

An error in the diagnosis of an oral or maxillofacial lesion could potentially be detrimental to a patient’s prognosis and management. Major discrepancies between the initial and subsequent diagnoses of head and neck pathologies range from 7 to 53%. This study determined the rate of discrepancies found in the diagnoses of oral and maxillofacial lesions after a second opinion in Saudi Arabia.

**Methods:**

A retrospective single-center study was conducted by oral and maxillofacial pathology consultants to review all cases referred for a second opinion to the oral and maxillofacial pathology laboratory between January 2015 and December 2020. If the second-opinion diagnosis matched the original diagnosis, this was described as “agreement.” If the second-opinion diagnosis did not match the original diagnosis but would not change the management or prognosis of a patient, this was classified as a “minor disagreement.” If the second-opinion diagnosis resulted in the changing of a patient’s management or prognosis, this was categorized as a “major disagreement.” Chi-square test and Fisher’s exact test were used to compare data between original and second-opinion diagnoses. A *p*-value of less than 0.05 was considered significant.

**Results:**

Of 138 cases, 59 (43%) had an initial diagnosis and a second-opinion diagnosis that were in major disagreement. The most common tumor for which there was a major disagreement was squamous cell carcinoma. No single factor influenced the occurrence of major disagreements.

**Conclusions:**

Our evaluation reiterates the importance of obtaining a second opinion from a specialist in oral and maxillofacial pathology to improve the diagnostic accuracy for lesions. A formal system for this step, in addition to the obtaining of adequate clinical and radiographic information about a patient, is mandatory for the review of difficult cases.

**Supplementary Information:**

The online version contains supplementary material available at10.1186/s12903-023-03085-w.

## Introduction

Several research studies have shown that lesions located in the head and neck, including oral and maxillofacial lesions, are highly prone to diagnostic errors due to the lesions’ complexity and the fact that general pathologists have minimal exposure to such lesions because of the lesions’ limited numbers [1–3]. The histopathological diagnosis of lesions of the head and neck region are ranked third in complexity after lesions of the female reproductive system and gastrointestinal lesions [4].

A second opinion in the field of pathology is defined as the reevaluation of a pathological diagnosis for outside cases by a second pathologist or a subspecialist pathologist experienced in a particular field. Usually, a patient, a pathologist, or a clinician requests a second opinion for several reasons, such as the uncertainty of a diagnosis, the rareness or difficulty of certain cases, or the wish to complete a prerequisite workup to finalize a diagnosis. A second opinion can be given retrospectively after an original diagnosis if an unexpected treatment outcome is observed [4].

Major discrepancies between initial and subsequent diagnoses of head and neck pathologies have been reported over the years, with the extent of discrepancy ranging from 7 to 53% [2, 3, 5]. Such disagreements imply that patients may have been receiving suboptimal treatment that could result in unfavorable outcomes [1–3, 6]. Therefore, consulting another pathologist for a second opinion is mandatory to reduce diagnostic errors and minimize their potential harm to patients [3]. This reevaluation could also reduce the resources spent on incorrect treatments [6], change patient management, and improve the overall patient care [2, 7].

Oral and maxillofacial pathology is defined by the American Dental Association as “the specialty of dentistry and discipline of pathology that deals with the nature, identification, and management of diseases affecting the oral and maxillofacial regions.” It is a recognized specialty of dentistry and pathology [8]. Practices in oral and maxillofacial pathology vary among countries. They are established in dental or medical schools, university hospitals, and tertiary medical hospitals. There are a limited number of oral and maxillofacial pathologists in different countries, and consequently, most pathological specimens from patients are reviewed by anatomical pathologists or pathologists in other specialties [9, 10]. There exist only a limited number of observational studies that compare an initial diagnosis from a referred case with a second diagnosis of oral and maxillofacial lesions [2, 3, 5, 7]. This makes it difficult to impossible to assess the potential impact of a second diagnosis on the management and prognosis of cases. This study aimed to determine the rate of discrepancies in the diagnosis of oral and maxillofacial lesions after a second opinion was obtained in a single academic hospital in Saudi Arabia.

## Materials and methods

### Study design and setting

This study was a retrospective single-center study conducted by oral and maxillofacial pathology consultants at the King Abdulaziz University Faculty of Dentistry (KAUFD) and the University Dental Hospital, Jeddah, Saudi Arabia. The faculty provides specialized dentistry services and is equipped with laboratories for pathological testing. The Research Ethics Committee approved the KAUFD proposal for this study and its conduct of this study (Proposal #043-05-20).

### Study population

The study population included all referred consultation cases with complete documentation retrieved from the oral and maxillofacial pathology laboratory database between January 2015 and December 2020. We excluded cases for which original reports were not available from our sample.

### Study procedures

Two consultants certified with the American Board of Oral and Maxillofacial Pathology (NB and YM) separately reviewed all of the cases retrospectively. For each case, these pathologists had access to a hematoxylin-eosin (H&E)–stained slide (or slides), special stains, immunohistochemistry (IHC), in situ hybridization (ISH) slides and/or a formalin-fixed paraffin-embedded tissue block specimen with an accompanying original pathology report and corresponding second-opinion diagnostic report. After comparing the original reports with the second-opinion diagnoses, cases were classified based on whether there was agreement, minor disagreement, or major disagreement between the two reports. If the second-opinion diagnosis matched the initial diagnosis, this was described as “agreement.” If the second-opinion diagnosis did not match the original diagnosis but did not result in a change in a patient’s management or prognosis, or if the report of the original diagnosis included only a histological description without a final diagnosis, this was classified as a “minor disagreement.” However, if the second-opinion diagnosis differed from the original diagnosis to the extent that it resulted in changing a patient’s management or prognosis, this was categorized as a “major disagreement.” In our study, changes in management or prognosis are primarily determined by pathological diagnosis, such as changes in disease category, cancer differentiation, or histological type of the same category.

If there was a disagreement in the classifications by the two reviewing pathologists (NB and YM), a third board-certified oral and maxillofacial pathologist (MA) reviewed the cases blindly and decided to which category the lesion should be assigned and consensus of all three consultants were considered.

### Data collection

Patient demographic and clinical data were obtained from patient databases. These data included the patient’s age and gender, the type of medical facility in which the patient had been diagnosed, the patient’s documented relevant clinical and radiographic information plus pathology reports, the biopsy site and tissue type of the lesion, and any ancillary tests used by the researchers in our study to reach a diagnosis. The data collected included information from both the original pathology report and second-opinion report.

### Statistical analysis

Categorical data were summarized as frequencies and percentages and the effect of different factors in agreement/disagreement was analyzed. We classified the type and nature of the diseased tissue based on the World Health Organization (WHO) classification of head and neck tumors [11]. Cases with diagnoses classified as in agreement were combined with diagnoses classified as minor disagreement and all were then described as in agreement. The chi-square test and Fisher’s exact test were done to compare the patient characteristics in cases in which a major disagreement existed between the original and second-opinion diagnoses. A *p*-value of less than 0.05 was considered significant. Data were coded and analyzed using the Statistical Package for the Social Sciences (SPSS) Windows version 22 (IBM Corporation, Armonk, NY, USA).

## Results

### Demographic and clinical characteristics

**Fig. 1:**
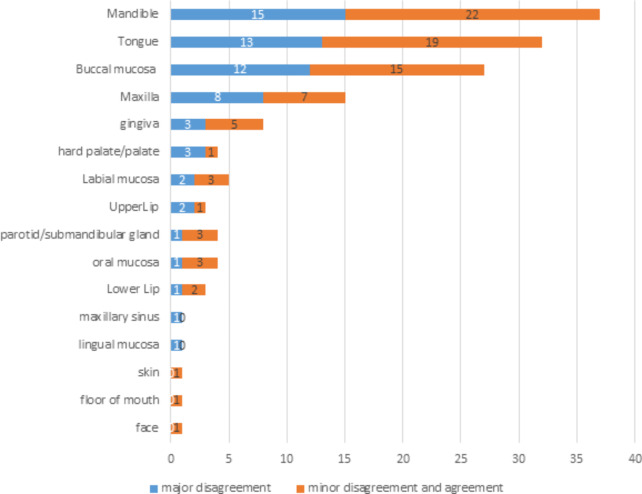
Site of biopsy by disagreement status for cases evaluated with a second opinion

There were 138 cases included in the study. The majority (35%) were younger than 35 years of age. The mean age was 44.4 years, and the range of ages was 2 to 90 years. Most patients were males (52%). Most of the cases were referred from government institutions (66%), and many of the 138 cases had associated relevant clinical information (83%) and radiographs (73%). Most of the biopsies were incisional (70%) and from soft tissues (84%). Of all cases, 83% were diagnosed using routine H&E staining. Only 24 cases (17%) required ancillary testing including special stains, IHC, and ISH; and 13 of those had discrepancies between the two diagnoses (Table 1). The most common site of biopsy disagreement was the mandible, followed by the tongue, buccal mucosa, maxilla, gingiva, and hard palate (Fig. 1).

**Table 1:** Characteristics of cases with major disagreement, minor disagreement, and agreement

Patient Characteristics	Total Number N = 138	Major Disagreement 59 (43%)	Minor Disagreement 19 (13%)	Agreement 60 (44%)	*p*-value*
Mean age (range) years	44 (2‒90)	45 (12‒90)	47 (10‒80)	86 (2‒86)	
Age group	Mean (%)				
<35 years	48 (35)	20 (42)	6 (13)	22 (46)	0.771
35‒55years	47 (34)	22 (47)	5 (11)	20 (42)	
55 + years	43 (31)	17 (40)	8(18)	18 (42)	
Sex					
Female	66 (48)	30 (45)	7 (11)	29 (44)	0.607
Male	72 (52)	29 (40)	12 (17)	31 (43)	
Type of institution					
Government	91 (66)	41 (45)	10 (11)	40 (44)	0.473
Private	47 (34)	18 (38)	9 (19)	20 (43)	
Relevant clinical information					
Yes	115 (83)	51 (44)	15 (13)	49 (43)	0.491
No	23 (17)	8 (35)	4 (17)	11 (48)	
Radiograph^±^					
Present	29 (73)	13 (45)	4 (43)	12 (41)	1
Absent	79 (27)	36 (46)	9 (11)	34 (43)	
Type of biopsy^±^					
Excisional	39 (30)	19 (49)	4 (10)	16 (41)	0.445
Incisional	93 (70)	38 (41)	12 (13)	43 (46)	
Type of tissue^±^					
Soft	112 (84)	46 (41)	16 (14)	50 (45)	0.634
Hard and soft^±^	21 (16)	10 (48)	2 (9)	9 (43)	
Required stains					
Yes	24 (17)	12 (50)	1 (4)	11 (46)	0.499
No	114 (83)	47 (41)	18 (16)	49 (43)	

*Chi-square statistics or Fisher’s exact test

^±^ Cases for whom information was missing

### Agreements and disagreements between original and second-opinion diagnoses

The second-opinion diagnosis was similar to the original diagnosis in 60 cases (44%), and the two diagnoses were therefore considered to be in agreement. The two diagnoses of 19 cases (13%) were considered to be in minor disagreement. The second opinion was the definitive diagnosis in 59 cases (43%) where there were major disagreement between the two diagnoses (see Table 1).

In summary, the two diagnoses of 59 cases (43%) were in major disagreement, that is, the second-opinion diagnosis would necessitate a change in the patient’s management or prognosis. For 79 cases (57%), the two diagnoses either were in agreement or were in minor disagreement but the second-opinion diagnosis would not change the patient’s management or prognosis (see Table 1).

### Major disagreements

The most common major disagreements based on the WHO tissue type and nature of a lesion were associated with lesions that were epithelial in nature (n = 24). Other cases of major disagreements in this study included 10 bone lesions, 7 soft tissue lesions, 6 immune-mediated conditions, 5 odontogenic tumors and cysts, 5 salivary gland diseases and tumors, 1 hematopoietic malignancy, and 1 sinonasal lesion. In 45 of the cases, there were no differences between the tissue type identified at the initial reading and the subsequent reading. In 14 of the cases, there were changes in both readings. Additionally, 12 cases with tumors had a change in diagnosis from reactive/benign to malignant and 2 cases had a change from malignant to benign (Supplementary Tables 1, Additional File 1).

**Fig. 2:**
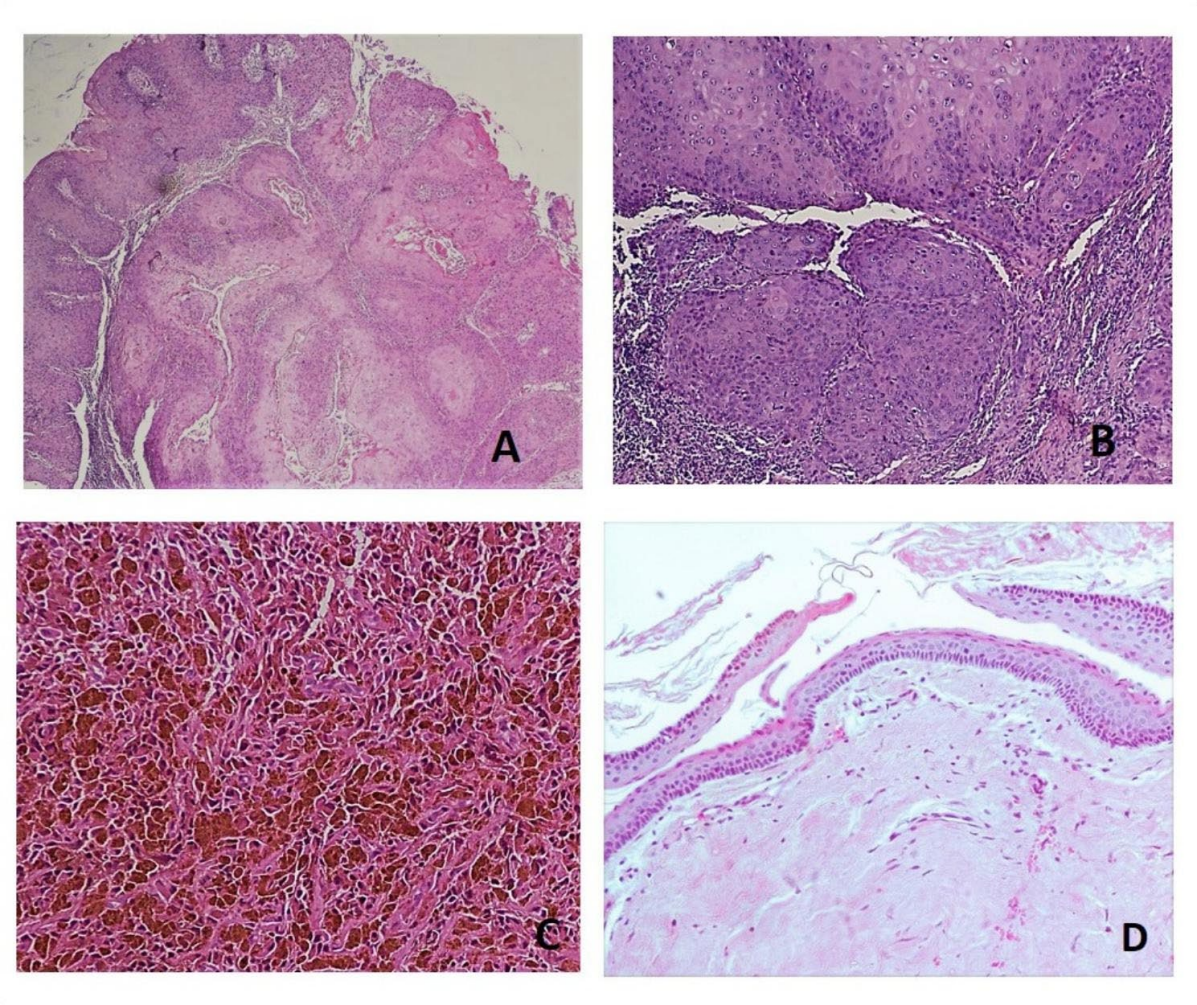
(**A**) SCC misinterpreted as verrucous carcinoma. The histomorphology showed verrucous configuration at H&E, 4X, but at higher magnification (**B**), showed invasive islands with dysplastic changes. (**C**) Malignant melanoma misinterpreted as blue nevus. Nests of spindle and epithelioid-like cells infiltrated the mucosa with abundant melanophages (H&E, 10X). (**D**) Odontogenic keratocyst misinterpreted as dentigerous cyst (H&E, 20X)

Of the 45 cases indicated above, 31 had changes in diagnoses, either to reactive lesions (cases 8, 15, 20, 28, 37, 50, 55, 60, 61, 66, 68, 69, 74, 75, 76, 77, 79, 89, 92, 93, 95, 96,109), benign lesions (cases 4, 71, 98), potentially malignant disorders (cases 5, 29, 45, 112, 123), or immune-mediated lesions (case 108). Seven had changes from a nonspecific diagnosis to a definite diagnosis (cases 38, 44, 47, 63, 91, 106, 132). For 1 case, the diagnosis was not conclusive and a second biopsy was recommended (case 138). In 5 cases, the histological type changed: in 1 case, the type of carcinoma changed from verrucous carcinoma to squamous cell carcinoma (case 136) (Fig. 2A and B). In 2 other cases, the malignant type changed after consultation with soft tissue pathologists and hemopathologists respectively; in case 10 it changed to spindle cell sarcoma (rhabdomyosarcoma, IHC strongly positive for muscle markers desmin and myoD1; negative for EMA, MART1, and CD99), and in case 110 it changed to lymphoma with further investigation is needed for typing (IHC: positive for CD3, CD2, and CD 20; negative for CD7 and EBV-encoded RNA). In 2 cases of benign tumors, the histological type changed to ameloblastoma (case 31) and osteoblastoma (case 78).

Of the 12 cases with changes from benign to malignant, squamous cell carcinoma and its variants were the most frequently mistaken malignancies in our study (n = 8; cases 34, 121, 128, 136, 82, 115, 122, 36). Other malignancies missed in the original diagnosis were salivary gland malignancies (n = 2; cases 56 and 22), osteosarcoma (n = 1; case 6), and oral melanoma (n = 1; case 101) (see Fig. 2C). In 2 cases, the diagnoses were changed from malignant to reactive or benign; they were severe mucositis (case 11) and pleomorphic adenoma (case 104).

Major disagreements were more frequent in patients 35 to 55 years of age and female patients. They were frequent even if there was relevant clinical information. Major disagreements were more frequent for cases that had excisional biopsies of both soft and hard tissue and cases for which radiographs were not provided. However, these factors were nonsignificant statistically (see Table 1).

### Minor disagreements

The most common lesions for which minor disagreements occurred based on WHO tissue type and nature were in epithelial tissue (n = 13). In cases of minor disagreement, a similar tissue type and nature of a lesion were observed at the initial and subsequent readings (Supplementary Tables 2, Additional File 1).

## Discussion

We set out to evaluate discrepancies between original and second-opinion pathological diagnoses for oral and maxillofacial lesions referred to a specialized center in Saudi Arabia. We found a major discrepancy rate of 43%. The most common type of malignant tumor associated with major disagreements was squamous cell carcinoma. There were no specific factors that influenced the rates of disagreement.

In this retrospective study, we found a high frequency of disagreement in the original and second-opinion diagnoses of the oral and maxillofacial pathology of lesions (56%). Of the 138 cases in our study referred for a second opinion from consultants who subspecialized in oral and maxillofacial pathology, there were major disagreements for 43% of cases and minor disagreements for 13% of cases. In the United States, a study of 142 cases found major disagreements for 16.3% and minor disagreements for 17.8% [3]. In the literature, the disagreement rate after a second-opinion histopathological diagnosis of an oral and maxillofacial or head and neck lesion ranged from 7 to 53%; this rate emphasizes the importance of obtaining a second-opinion diagnosis from an oral and maxillofacial pathologist [2, 3, 5, 6].

The original diagnoses were mostly made by general pathologists or pathologists who had specialized in fields other than oral and maxillofacial pathology. These pathologists and specialists do not routinely see many of these lesions because the lesions are uncommon, and therefore, their experience in diagnosing the lesions is limited. Most of these cases are sent to oral and maxillofacial pathologists [3]. Furthermore, there are still no uniform, dependable referrals to a specific subspecialty for pathological diagnoses, including diagnoses of oral and maxillofacial pathological lesions [10]. Also, some pathologists may not be familiar with the histoanatomical features and classifications of lesions in the oral and maxillofacial regions [4]. For instance, the histological features that could identify types of odontogenic cysts and tumors may be difficult to detect, especially if lesions are inflamed (see Supplementary Tables 1, Additional File 1; cases 20, 55, 69; see Fig. 2D) or are infrequently encountered by a pathology service [12]. Although most referred specimens had not been stained by immunohistochemical or special stains, only 24 cases (17.4%) referred for the study’s second-opinion diagnoses required staining; 12 of these had major disagreements in diagnosis, and 1 had a minor disagreement. Nevertheless, most pathological diagnoses of oral and maxillofacial lesions rely mainly on histomorphological findings. Sometimes, a confirmatory special stain and immunohistochemical panel are required to specify the tissue type and assign a lesion to a specific category [13, 14]. The fact that these ancillary tests were not used at referring facilities may be interpreted as an absence of advanced diagnostic services at these institutes or as the unfamiliarity of the pathologists with these ancillary tests [1, 14–16]. Thus, there is a need for specialized services and specialist pathologists for the diagnosis of oral and maxillofacial lesions.

Histopathological evaluations of the oral cavity and jaws depend mainly on the clinical information and radiographic findings for bony lesions [3, 13]. However, this information was either missing or insufficient to support a histopathological evaluation for the cases in our study. For example, a definite diagnosis of osteitis fibrosa cystica (see Supplementary Tables 1, Additional File 1; case 37) is only possible when a patient’s clinical data reveal a history of hyperparathyroidism, and a sinus mucocele is mainly diagnosed based on clinical and radiographic findings (see Supplementary Tables 2, Additional File 1; case 66). It is essential to have adequate supporting information to reduce diagnostic errors by oral and maxillofacial pathologists.

The distribution of discrepancies in the cases in our study depended on the histological tissue type. Commonly, disagreements were seen in epithelial tissue (47%; n = 37/79). Similar results were seen in a previous study in which dysplasia and squamous cell carcinoma were the most common second-opinion diagnoses [3]. The second review changed the diagnosis from reactive or benign to malignant for 12 cases, reactive or benign to potentially malignant disorders for 5 cases, and reactive to benign for 3 cases. The most frequent malignant second-opinion diagnosis was squamous cell carcinoma (8 cases); this was possibly due to the fact that squamous cell carcinoma is the most common malignant tumor of the oral cavity [15].

We did not find any specific factors that may have influenced the occurrence of major disagreements. In our cohort, there was a higher frequency of disagreement for female patients; this contradicts a previous study by Zhu et al. where the frequency of disagreement was 61% for males. However, in both this study and the previous study, the association between gender and discrepancy rates did not attain statistical significance [7]. It is possible that our sample, which was mainly based on referrals from clinicians due to their concern about the original diagnosis, may have been biased or insufficiently powered to generate specific conclusions. Pending further larger representative reevaluations, our results suggest that all samples of head and oral and maxillofacial lesions ought to undergo a second review because it may lead to changes in management and improve patients’ care [2, 8].

The main strengths of the study included its location in a specialized facility that is central for the referral of oral and maxillofacial lesions. The study had a representative sample population drawn from both governmental and private centers. A limitation of this study may be that it may have overestimated the disagreement rate, since most cases included in the study were referrals from a surgeon or clinician who was seeking a second opinion that would either confirm or refute the original diagnosis before starting patient management. In this retrospective analysis, the clinical and radiographic information available was limited to that provided in requisition forms.

Our study on the provision of second-opinion consultations for oral and maxillofacial pathology led to nearly half of the cases having a change in management and prognosis. Thus, we reiterate the importance of a second opinion from a specialist in oral and maxillofacial pathology to improve the diagnostic accuracy. A formal system for obtaining a second-opinion pathology diagnosis from a subspecialist offers an excellent opportunity for the review of difficult cases.

The adoption of telepathology using digitized slides for a second-opinion diagnosis may reduce diagnostic delays and make oral maxillofacial pathologists accessible worldwide [17]. Furthermore, since misdiagnoses are frequently related to lack of clinicopathologic correlation, a concerted team effort by clinicians to provide pathologists with adequate clinical information about a patient will facilitate the evaluation of specimens, which can limit diagnostic errors and ultimately improve patient care.

## Electronic supplementary material

Below is the link to the electronic supplementary material.

Supplementary Material 1

## Data Availability

The datasets used and/or analyzed during the current study are available from the corresponding author on reasonable request.
