# Supplementary material for: The value of a specialized second-opinion pathological diagnosis for oral and maxillofacial lesions
Source: BMC Oral Health. 2023 Jun 9;23:378. doi: 10.1186/s12903-023-03085-w (PMC10257276; doi:10.1186/s12903-023-03085-w)
Supplement: Supplementary file 1 — Supplementary Material 1 [file 12903_2023_3085_MOESM1_ESM.docx]

**Supplementary Table 1.** Description of cases where there were major disagreements between the original and second-opinion diagnoses*****

| **Case ID** | **Tissue Type Based on WHO First Reading** | **Original Diagnosis** | **Tissue Type Based on WHO Second Reading** | **Second-Opinion Diagnosis** |
| --- | --- | --- | --- | --- |
| 4 | Bone | Chronic inflammation with reactive lymphoid follicles | Bone and odontogenic tumor | Foreign body reaction with granulation tissue and odontoma |
| 5 | Epithelium | Acanthosis, lymphocytes/ squamous hyperplasia with suggestive irritation change; negative for malignancy or dysplasia | Epithelium | Mild dysplasia/ hyperkeratosis with epithelial atrophy |
| **6** | **Bone** | **BFOL with cementoma** | **Bone** | **An atypical bone tumor** |
| 8 | SG | Mucocele | Soft tissue | Parulis |
| 10 | Bone | Osteosarcoma | Soft tissue | Spindle cell malignancy (rhabdomyosarcoma) |
| 11 | Epithelium | Moderately differentiated infiltrating SCC, muscle infiltration, perivascular invasion | Epithelium | Severe mucositis |
| 15 | Soft tissue and bone | Inflammation non-tuberculosis | Soft tissue | Vascular hamartoma with fibrosis |
| 20 | Odontogenic cyst | Inflamed dentigerous cyst | Odontogenic cyst | OKC |
| **22** | **SG** | **Oncocytoma** | **SG** | **Acinic cell carcinoma of parotid gland** |
| 28 | Bone | Ossifying fibroma | Bone | BFOL consistent with cemento-osseous dysplasia, florid type |
| 29 | Epithelium | Buccal: Chronic spongiotic dermatitis  Tongue: consistent with lichen planus | Epithelium | Buccal: Keratosis of unknown significance  Tongue: mild dysplasia, tongue: moderate dysplasia |
| 31 | Odontogenic tumor | Ameloblastoma (unicystic), tooth #46: positive margin of ameloblastoma | Odontogenic tumor | Ameloblastoma, tooth #46: fibrosis with chronic inflammation |
| **34** | **Epithelium** | **Fragments of fibrovascular tissue covered by reactive squamous epithelium with necrosis and active inflammatory processes, degenerative bone, fibrosis and fibrovascular with chronic inflammation** | **Epithelium** | **SCC** |
| **36** | **Epithelium** | **Hyperkeratosis and parakeratosis consistent with leukoplakia** | **Epithelium** | **Well-differentiated SCC** |
| 37 | Bone | Consistent with extramedullary haematopoiesis | Bone | Osteitis fibrosa cystica |
| 38 | Bone | Fibrosis with chronic inflammation and eosinophilic microabscess | Bone | Chronic osteomyelitis |
| 44 | Epithelium | Mild pseudoepithelial hyperplasia with surface ulceration and granulation tissue with fibrosis; no malignancy | Epithelium | Traumatic ulceration granuloma with stromal eosinophilia with areas of spongiotic epithelium and hyperkeratosis |
| 45 | Epithelium | Squamous mucosa with mild inflammation; no evidence of malignancy | Epithelium and mesenchymal (soft tissue) | Submucosal fibrosis |
| 47 | Epithelium | Plasma cell granuloma | Epithelium | Plasma cell mucositis/ cheilitis and seborrheic keratosis |
| 50 | Bone | Bony tissue and fragment of necrotic tissue with purulent exudate | Bone | Viable bone and keratin elements |
| 55 | Odontogenic cyst | Dentigerous cyst | Odontogenic cyst | OKC |
| **56** | **SG** | **Myoepithelioma of minor SG (negative for malignancy)** | **SG** | **Cribriform adenocarcinoma of minor SG, buccal mucosa** |
| 60 | Epithelium | Aphthous ulcer | Epithelium | Acanthosis with mild chronic inflammation |
| 61 | Bone | Garre’s osteomyelitis | Odontogenic cyst | Inflamed odontogenic cyst |
| 63 | Epithelium | Nodular leukoplakia | Soft tissue | Fibroepithelial hyperplasia |
| 66 | Odontogenic cyst | Eruption cyst | Sinus cyst | Sinus mucocele |
| 68 | Epithelium | Hyperkeratotic, acanthotic stratified squamous cell epithelium; no dysplasia | IM | Lichen planus |
| 69 | Inflammatory odontogenic cyst | Residual cyst | Odontogenic cyst | OKC |
| 71 | Soft tissue | Pyogenic granuloma | Epithelium | Sinonasal papilloma |
|  |  |  |  |  |
| 73 | Epithelium | Verrucous carcinoma | Epithelium | Well-differentiated SCC |
| 74 | Epithelium | Pseudoepithelial hyperplasia with acanthotic hyperkeratotic squamous mucosa | Soft tissue | Vascular malformation |
| 75 | Bone | BFOL: Cemento-osseous dysplasia | Bone | Traumatic bone cyst |
| 76 | Epithelium | Acanthosis with parakeratosis and mixed inflammatory infiltrate | IM | Mucous membrane pemphigoid |
| 77 | Epithelium | Active chronic inflammation with inflammatory ulcer and mild dysplasia | Epithelium | Nonspecific ulcer (traumatic) |
| 78 | Bone | Calcifying epithelial odontogenic tumor | Bone | Aggressive (epithelioid) osteoblastoma |
| 79 | Epithelium | Inflammation with low-grade focal dysplasia, inconsistent with erythroplakia | Epithelium | Acanthosis, mild epithelial atypia with microabscesses consistent with geographic tongue |
| **82** | **Epithelium, IM** | **1: Hyperplastic epithelium with parakeratosis consistent with leukoplakia; 2: lichen planus; 3: atypical pseudoepithelial hyperplasia** | **Epithelium** | **1: Epithelial dyskeratosis consistent with benign intraepithelial dyskeratosis; 2: epithelial dysplasia; 3: well-differentiated SCC** |
| 89 | Epithelium | Active chronic inflammation with squamous hyperplasia (leukoplakia) and mild dysplasia | IM | Chronic mucositis consistent with lichen planus |
| 91 | SG | Inflamed extravasation mucocele | SG | Mucous extravasation phenomenon with actinomycosis and sialolith |
| 92 | Autoimmune | Oral discoid lupus | IM | Lichenoid mucositis with post inflammatory hyperpigmentation |
| 93 | Epithelium | Chronic nonspecific inflammation and parakeratosis | IM | Lichen planus |
| 95 | Epithelium and fungal infection | Inflamed and hyperplastic squamous epithelium, candidal infection | Epithelium | Nonspecific ulcer and chronic mucositis |
| 96 | Epithelial and mesenchymal (soft tissue) | Benign mucosal tissue with chronic inflammation | Soft tissue | Granulomatous inflammation |
| 98 | Bone | Giant cell reparative granuloma | Bone | BFOL consistent with juvenile ossifying fibroma |
| **101** | **Epithelial melanocyte** | **Blue nevus** | **Epithelial melanocyte** | **Melanoma** |
| 104 | SG | Adenoid cystic carcinoma | SG | Pleomorphic adenoma |
| 106 | Bone | Ossifying fibroma | Soft tissue | Peripheral ossifying fibroma |
| 108 | Epithelium | Keratosis, lichenoid with mild dysplasia | IM | Chronic mucositis and hyperkeratosis consistent with lichen planus |
| 109 | Bone | Osteoma | Bone | Fragments of viable and necrotic bone |
| 110 | Hematopoietic | Extranodal (angioinvasive) natural killer‒cell/T-cell lymphoma of palate | Hematopoietic | Atypical lymphoid proliferation with a nonspecific ulcer; further immunostaining is recommended |
| 112 | Epithelium | Hyperkeratosis consistent with leukoplakia | Epithelium | Mild dysplasia and hyperorthokeratosis |
| **115** | **Epithelial-mesenchymal** | **Atypical epithelioid and spindle cell infiltrate** | **Epithelium** | **Spindle cell malignancy favouring spindle cell carcinoma** |
| **121** | **Epithelium** | **Squamous hyperplasia with features consistent with wart virus infection** | **Epithelium** | **SCC** |
| **122** | **Bone** | **Central giant cell lesion** | **Epithelium** | **Spindle cell (sarcomatoid) carcinoma** |
| 123 | Epithelium | Wart/papilloma with wart virus | Epithelium | Papillary epithelial proliferation with mild atypia; re-biopsy is suggested due to poor orientation |
| **128** | **Epithelium** | **Benign lichenoid acanthosis/keratosis; no dysplasia; no malignancy** | **Epithelium** | **Superficially invasive SCC** |
| 132 | Epithelium | Ulcerative sialadenitis of minor SG | Epithelium | Ulceration lesion with EBV(+)/CD30(+) positive lymphoid infiltration and candidiasis; differential diagnosis includes EBV positive mucocutaneous ulcer (EBV-MCU) vs. EBV positive posttransplant lymphoproliferative disorder (further workup needed |
| **136** | **Epithelium** | **Verrucous lesion** | **Epithelium** | **SCC, well differentiated** |
| 138 | SG | Fibrous tissue, SG, and chronic inflammation | SG | Fat tissue with chronic inflammation; SG tissue with atypical cellular proliferation |

*12 cases whose diagnosis changed from benign to malignant are highlighted in bold.

BFOL, benign fibro-osseous lesion; EBV, Epstein-Barr virus; IM, immune-mediated lesion; OKC, odontogenic keratocyst; SCC, squamous cell carcinoma; SG, salivary gland.

**Supplementary Table 2.** Description of cases where there were minor disagreements between original and second-opinion diagnoses

| **Tissue Type Based on WHO First and Second Readings** | **Case ID** | **Original Diagnosis** | **Second-Opinion Diagnosis** |
| --- | --- | --- | --- |
| Soft tissue | 7 | Acute and chronic inflammation with fibroblastic proliferation and edema | Juvenile spongiotic gingival hyperplasia |
| Epithelium | 13 | Poorly differentiated invasive SCC | Moderately differentiated SCC |
| Epithelium | 14 | Acanthosis with parakeratosis | Nonspecific ulcer with chronic and acute inflammation |
| Epithelium | 19 | Two biopsies: well-differentiated SCC (no invasion) | 1: Superficially invasive well-differentiated SCC; 2: carcinoma in situ |
| Bone | 23 | Giant cell granuloma | Ameloblastic fibro-odontoma with CGC lesion |
| Epithelium | 27 | Poorly differentiated SCC | Well-differentiated SCC |
| Bone | 39 | Acute and chronic inflammation with granulation tissue and microabscess, suggestive of acute osteomyelitis | Chronic osteomyelitis |
| Odontogenic tumor | 41 | Ameloblastic fibroma | Complex odontoma |
| Epithelium | 51 | Superficial nonspecific active chronic inflammatory process with ulceration, suggestive aphthous ulcer | Mild inflammation with no ulceration |
| Epithelium | 54 | SCC | Poorly differentiated SCC |
| Epithelium | 59 | Oral melanoacanthoma | Multifocal diffuse pigmentation |
| Epithelium | 80 | Inflamed fibroepithelial polyp with leukoplakia | Hyperkeratosis with chronic inflammation |
| Epithelium | 87 | Plasma cell granuloma/chronic sialadenitis | Nonspecific ulcer |
| Epithelium | 88 | Oral lichenoid reaction | Lichen planus |
| Epithelium | 94 | Bullous pemphigoid | Hyperkeratosis and subepithelial clefting consistent with mucous membrane pemphigoid |
| Epithelium | 113 | Benign squamous mucosa with ulceration | Traumatic ulcerative granuloma with stromal eosinophilia |
| Epithelium | 114 | Lichen planus | Hyperkeratosis with lichenoid inflammation |
| Odontogenic cyst of bone | 131 | Inflamed granulation tissue | Radicular cyst, granulation tissue and tooth fragment |
| Soft tissue | 135 | Inflamed granulation tissue | Ulcerated peripheral ossifying fibroma |

CGC, central giant cell lesions; SCC, squamous cell carcinoma.
